# Cardiac Surgery-Associated Acute Kidney Injury: Current Updates and Perspectives

**DOI:** 10.3390/jcm11113054

**Published:** 2022-05-28

**Authors:** Christian Ortega-Loubon, Eduardo Tamayo, Pablo Jorge-Monjas

**Affiliations:** 1 Department of Cardiac Surgery, Clinic Hospital of Barcelona, Villarroel ST 170, 08036 Barcelona, Spain; 2 BioCritic Group for Biomedical Research in Critical Care Medicine, Ramon y Cajal Ave. 7, 47005 Valladolid, Spain; tamayo@med.uva.es (E.T.); pablojor@yahoo.es (P.J.-M.); 3 Department of Anaesthesiology, Clinic University Hospital of Valladolid, Ramon y Cajal Ave. 3, 47003 Valladolid, Spain

Cardiac surgery-associated acute kidney injury (CSA-AKI) is a recognized and serious complication and one of the stronger risk factors for mortality in patients undergoing cardiac surgery [1]. In its more severe stage, it increases the odds of death by an alarming 50–80% [2]. Even a slight increase in serum creatinine after cardiac surgery implies a higher morbidity, with a longer length of stay in both hospital and the intensive care unit as well as higher healthcare costs.

This Special Issue of the *Journal of Clinical Medicine* (*JCM*), entitled “Cardiac Surgery-Associated Acute Kidney Injury: Current Update and Perspectives”, offers valuable research articles that enhance our general understanding of an intricate scientific field, which involves the genetic susceptibility, incidence, and epidemiology of CSA-AKI; risk stratification; risk–model comparisons; diagnosis criteria; the importance of novel biomarkers; prevention strategies; treatment and management; patient outcomes; and economic impacts.

Molina et al. [3] pinpoints the importance of the Leicester Score to predict CSA-AKI, which was created in 2014 but barely used. They highlight that this relatively new model can predict CSA-AKI at any stage, and has better discrimination score (AUC 95% Confidence Interval (CI) 0.721 (0.671–0.771) *p* < 0.001) compared to Cleveland Clinic Score (AUC 95% CI 0.505 (0.54–0.65) p0.001) and Euroscore (AUC 95% CI 0.662 (0.611–0.713) *p* < 0.001).

Schurle et al. [4] examined the incidence and epidemiology, emerging biomarkers, treatments and bundles, patient outcomes, and economic impact of CSA-AKI. From the diagnosis of CSA-AKI, specific treatments are scarce and limited to standard supportive care. Nevertheless, novel biomarkers combined with care bundles are promising forms of treatment [5,6]. With respect to economic impacts, the cost of CSA-AKI is difficult to calculate given the different ways that it influences patient outcomes. Alshaikh et al. found that hospital costs were as high as USD 26,000 in patients with AKI not requiring renal replacement therapy (RRT) and exceeded USD 69,000 for AKI requiring RRT, leading to a total annual cost for CSA-AKI of almost USD 1 billion in the United States [7]. 

While the relationship between AKI and chronic kidney disease (CKD) was previously studied in the literature, this association has not yet been reported following cardiac surgery [8]. Choe et al. [9] showed that while transient stage 1 AKI was not significantly associated with CKD (hazard ratio (HR) 1.95, 95% CI 0.83–3.02, *p* = 0.246), persistent stage 1 AKI and transient and persistent stage 2 or 3 AKI showed significant associations with CKD (persistent stage 1: HR 3.11, 95% CI 2.62–4.91; transient higher stage: HR 4.07, 95% CI 2.98–6.11; persistent higher stage: HR 13.36, 95% CI 8.22–18.72, all *p* < 0.001). These authors also demonstrated that CSA-AKI lasting longer than 7 days was more strongly associated with CKD, confirming that both the duration and severity of CSA-AKI were of paramount importance for the development of CKD. They recommend that the kidney function of those who developed transient AKI should be followed up on the third day after the occurrence of AKI to monitor and determine whether persistent AKI develops. Moreover, the renal functions of those with persistent AKI should be followed up seven days after the onset of AKI to evaluate their tendency to develop CKD. 

Similarly, Massoth et al. [10] pinpointed the different stages of AKI, ranging from a mild injury to chronic kidney disease. This framework spectrum ranges from a subclinical KDIGO stage 1 to a persistent AKI state beyond 7 days, which falls into the category of acute kidney disease and disorders (AKD). Although recovery may occur at any point during this timeline, disease progression may also occur, eventually leading to a CKD if persistent for more than 3 months. In their article, these authors highlighted the crucial role that biomarkers play in the management of CSA-AKI as they can cause further subclinical damage and serve as additional discriminative tools.

In the review article regarding genetic susceptibility to AKI, the variety of genes acting collectively in the pathogenesis of this disease is particularly interesting. The most relevant genes are those related to inflammatory systemic responses, more specifically, TNF- α, which highlights the enormous impact that this response has on the pathophysiology and etiology of this entity. Genetic variants may affect the kidney reaction to a noxious stimulus, ranging from a mild injury to a more severe condition.

Since the pathophysiology of CSA-AKI is multifactorial, its prevention, and treatment is far from simple. This is revealed in the review article by Osterman et al., which reveals that, to date, there remain no specific pharmacological therapies for CSA-AKI [11]. These authors wisely summarize the perioperative strategies to reduce the risk of CSA-AKI. Prevailing evidence supports a multimodal risk-stratified approach, involving goal-directed perfusion, biocompatible coatings during cardiopulmonary bypasses (CPBs), and biomarker-guided postoperative management based on the KDIGO care bundle recommendations. 

Despite all these recent advances, we must continue to improve both our general understanding and capacity to effectively diagnose, treat and/or prevent CSA-AKI, as it remains a widespread concern in clinical medicine and remains a threat to postoperative cardiac patients. This Special Issue of the *Journal of Clinical Medicine*, entitled “Cardiac Surgery-Associated Acute Kidney Injury: Current Update and Perspectives”, not only aims to provide an updated and comprehensive summary of the clinical approaches to this disease, in which a variety of factors are intertwined, but also to introduce the latest advances in diagnosis, treatment, and prevention strategies in order to effectively tackle this issue.

## Data Availability

Not applicable.
